# Moving from Outsider to Insider: Peer Status and Partnerships between Electricity Utilities and Residential Consumers

**DOI:** 10.1371/journal.pone.0101189

**Published:** 2014-06-30

**Authors:** Peter Morris, Laurie Buys, Desley Vine

**Affiliations:** 1 School of Design, Creative Industries Faculty, Queensland University of Technology, Brisbane, Australia; 2 School of Electrical Engineer and Computer Science, Science and Engineering Faculty, Queensland University of Technology, Brisbane, Australia; Tianjin University of Technology, China

## Abstract

An electricity demand reduction project based on comprehensive residential consumer engagement was established within an Australian community in 2008. By 2011, both the peak demand and grid supplied electricity consumption had decreased to below pre-intervention levels. This case study research explored the relationship developed between the utility, community and individual consumer from the residential customer perspective through qualitative research of 22 residential households. It is proposed that an energy utility can be highly successful at peak demand reduction by becoming a community member and a peer to residential consumers and developing the necessary trust, access, influence and partnership required to create the responsive environment to change. A peer-community approach could provide policymakers with a pathway for implementing pro-environmental behaviour for low carbon communities, as well as peak demand reduction, thereby addressing government emission targets while limiting the cost of living increases from infrastructure expenditure.

## Introduction

Internationally, peak electricity demand is an energy policy concern with the substantial expenditure in updating transmission, distribution and generation infrastructure adding to the cost of living while only being required during a handful of days per year. Residential loads contribute significantly to seasonal and daily peak electricity demand [1] and account for approximately one third of the total peak electricity demand [2]. One of the most immediate options for lowering peak electricity demand is to focus on electricity use by residential consumers, since peak reduction only requires the desire of residential consumers to change electricity use behaviour [3]. There are few academic studies that investigate successful residential energy behaviour change across a sizable community over a prolonged period, with OPower [4] and Juneau [5] being important examples. Another such example, which forms the basis of the paper, is a project successful over an extended timeframe in reducing peak electricity demand levels across an entire Australian island community of around 2200 permanent residents. This paper reviews this notable successful project through the perceptions and experiences of island residents regarding the community engagement and relationship development activity undertaken during the project and the effect of this activity on peak demand reduction.

### Approaches to electricity use behaviour change

Peak electricity demand reduction programs aim to reduce total peak electricity demand or shift demand from peak to off-peak times without substantially affecting the consumer. These programs can be achieved through policies such as incentives and consumer education [6]. Government law, regulations and incentives encourage specific positive behaviours and/or discourage specific negative behaviours. To date, policies have tended to rely on punishments such as penalties and taxes, or rewards such as rebates and incentives to change behaviour [7].

Successful policy design is usually based on interpreting individual behaviour [8], understanding people's cultural practices, as well as social and community interactions [9]. While time-dependent pricing schemes, including dynamic pricing, time-of-use pricing, peak pricing and critical peak pricing, have all influenced consumer behaviour [10], there is still doubt about the long term impact of implementing pricing schemes in a large population. There is no consensus that consumers will react appropriately or which pricing plans could be adopted to affect behaviour [11]. Also, the removal of interventions can influence continued behaviour change [12] including those based on financial incentives [13]. Similarly, education that has highlighted financial incentives for electricity use behaviour change has failed to fulfil its potential of long term peak electricity demand reduction [14] and targeting attitudes through education or the use of financial incentives has been shown to affect only short term behaviour [15].

Education programs mostly describe the nature and severity of the problem in an effort to change people's attitudes, or outline specific actions individuals can take to help solve the problem [16]. These programs are typically run via television, newspapers, books, magazines and other media outlets to convince the public that action on their part is essential [7]. Like education programs, information programs appear to have little impact on behaviour change [17], and education alone does not lead to electricity demand reduction [18], [19]. Information programs depend on how the information is conveyed [20] and must be specific enough to effect behaviour change [21], by including behavioural factors during the policy design phase [20]. The failure of these informational programs to foster behaviour change is due in part to developers underestimating the difficulty of changing behaviour [14].

Many field studies have investigated behaviour change strategies [18] and these have usually fallen under the category of information, feedback, goal setting or rewards. With this research having been undertaken in a number of countries using different methodologies (including numbers of participants in trials and duration of the study) the results reflect a mix of promise [22], [23], [24] and a need for caution [12], [25], [26]. Some studies show considerable savings while others indicate a spread of results over a wide range.

Energy assessments in residential households (sometimes called home energy audits) have shown encouraging results in electricity demand reduction [17], [23]. An energy assessment is a process where the house is inspected and analysed, with the goal of identifying opportunities for reducing energy use without impacting the lifestyle of the residents [27]. The lower electricity demand, after an energy assessment has been performed, is likely due to information being directly tailored to the consumer and house design. Nevertheless, few consumers request energy assessments, even when free, suggesting that only the most motivated consumer will embrace this type of intervention [28]. In more recent research, less than 10% of customers request or perform energy assessments, despite heavy marketing, promotional pricing and a variety of energy assessment styles offered [29]. Only when a majority of households, and not just those few motivated consumers who are orientated to behaviour change, request energy assessments, would it be possible to assess the full potential of using this type of intervention for reducing peak electricity demand.

### Value of Community Focus

Focusing at the community level, rather than at the individual consumer level, may bring greater success to changing residential electricity use behaviour [30], [31]. Achieving participation by a large number of residential consumers within a community context, requires an approach which fosters social cohesion and community engagement [32]. An early step in community engagement is being easily accessible, and communicating with the target audience, while listening carefully [33]. Empathy built from regular communication may facilitate behaviour change or in general, the adoption of a new idea [34]. A key element of successful partnerships is that they concentrate on the human element of the relationship rather than the financial one [35]. A successful community partnership is built on alliances that work towards a common purpose [36]. Being able to depend on another party is a strong indicator of trustworthiness [37], which is a basic cornerstone in building a successful community partnership [33].

Sometimes government and business can be seen as untrustworthy due to non-shared goals and benefits with the community on energy efficiency priorities [16] and trust in energy utilities specifically can be low [38]. An approach which increases trust in the information source would be beneficial for changing energy use behaviour. Government department and energy utility success in the community seems built on local rather than detached entities, and trust must be consistently accepted for long term change to be possible [7]. Communities offer a safe environment for information and experience to transfer within the social fabric [39], and present the opportunity for enlisting individuals into group objectives for the improvement of behaviours for a common goal [40]. The social group creates a community where behaviour change can be precipitated by a leader with interest and a voice for change [41].

The community leader, being “persons to whom others look for advice or a role model to emulate regardless of his or her official status” [42], may bring knowledge or views which can influence the community toward action and change [43]. This heightened awareness of a particular issue through strong community involvement can exert pressure to change individual electricity use behaviour, particularly when economic and environmental goals are clear [44]. People look for ways to manage the constant stream of data available in the current information age [45] by considering thoughts and views from others within their social circle. These social networks can reinforce behaviours into strong habits which then become difficult to change [46].

Given that electricity use is at the core of everyday living [47] and human learning is best done in a social environment [48], the community can be the education vehicle for electricity use behaviour change. Learning in a social group avoids the need for trial and error, with respected community peers providing strong attention and motivation for similar behaviours within the group [49]. A peer is a person who shares related values, experiences, and lifestyle [50] or a person of equal worth or status [51]. Peer acceptance refers to the building of strength in the relationship, in the same way that friends have a mutual bond. Peers can legitimise knowledge with, information from someone known, such as a peer, being more likely to be acted upon [52]. Peers and community leaders tend to transfer information or knowledge between groups of people across boundaries [34]. Therefore any member of a social group, who has been accepted as trustworthy and credible on a particular topic, can educate and influence the actions of the other members of the social group. Thus, it could be argued that by becoming a peer, the energy utility is better positioned to change residential electricity use behaviour.

### Context of the current study

The current case study reviews a project which was exceptionally successful in firstly, attaining requests for home energy assessments from over 80 percent of households and secondly, in achieving significant energy conservation and peak demand reduction across an entire island community (see Appendix S1 for the background and description of the project and Figures S1 and S2 included in Appendix S1 for graphical representation of the successful outcomes in the reduction of daily peak demand and annual grid supplied electricity consumption) off the Australian Queensland coast. Peak electricity demand was not only abated but has continued to reduce during the project period so that peak electricity demand has fallen below pre-intervention levels (see Figures S1 and S2 in Appendix S1). While the case study project used solar photovoltaic panels as part of the suite of interventions, solar photovoltaic panels made no impact on peak demand reduction due to the timing of peak being after sunset (see Appendix S1).

The rarity of investigating a successful long term peak demand reduction program, particularly from the perspective of the consumer, provides an internationally relevant case study to inform policy and practice directions. Personal contact has been found to create a trusting relationship between industry and households [53], and trust strengthens between groups the longer the relationship develops [54]. Establishing a relationship of mutual benefit by becoming a member of the community through engagement is conducive to achieving goals [55]. Thus, the purpose of this article is to investigate the experience of the residential consumers, and their perceptions of the relationship between the energy utility, community and themselves, and how the energy utility was able to elicit broad-ranging support to achieve community-based outcomes. This investigation will provide insight into the community engagement activities, as perceived by the residential consumers, to establish a relationship favourable to achieving peak demand reduction.

## Method

This research is part of a larger study addressing peak electricity demand. This particular article examines and reports on one section of the data exploring peer status and partnership between an energy utility and individuals in a community. This research is based on a phenomenological, qualitative approach, which prioritises participants own words and voices in expressing and understanding their day to day lived experiences. The main reason for conducting a single case study was to investigate a unique case, with the advantage of high discoverability [56]. In-depth insight into issues and topics and an exploration into the social and cultural contexts that affect processes, decisions and events, were explored through the real life experiences of Magnetic Island residential consumers who participated in the Townsville Solar City Project. Ethical approval for this project was obtained from the Queensland University of Technology University Human Research Ethics Committee (UHREC), consisting of a Chairperson with suitable experience and senior academic standing, one person who performs a pastoral care role in the community, representation from each faculty within the university, one lawyer, two lay people (one man and one woman), a medical practitioner, a counsellor, and one nominee from the Aboriginal and Torres Strait Islanders Committee. In addition, all participants provided written informed consent prior to their participation in the current study.

This research used qualitative, in-depth interviews to gather specific information based on participant descriptions of their everyday household experiences [57]. Qualitative research does not suggest generalisation of the results across populations. The purpose of qualitative methodology is to collect data that illuminates the phenomena under study. Thus a deeper understanding is obtained from the full and meaningful responses provided by the participants [58]. In qualitative research, sample size is less important as the priority is the creation of patterns and themes that accurately represent meaning. Qualitative research is an internationally recognised and rigorous social research method that is used to gain in-depth knowledge and understanding of a particular issue or question.

### Participants

A total of 30 participants (18 Females and 12 Males) from 22 Magnetic Island households were selected from local community sources including a local resident data base, energy utility customer database and local contacts. Household size varied from one resident to seven residents, with the majority being two residents per household. Participants selected were from key community resident types including single working people (four), working couples (ten), house share (one), retirees (five) and families with children (two). Ages ranged from early 30's to late 70's with most of the participants being in the 45 to 65 age range. All participants were permanent residents of Magnetic Island. Nearly two thirds were employed full time either on the Island or in nearby Townsville.

### Procedure

Interviews gathered data about the participants' initial experiences and ongoing family adaptations to changing behaviour for electricity use. The semi-structured interview format enabled residents to provide an in-depth understanding of their experiences from their perception. The interviews were conducted in participant homes or at a convenient location and lasted approximately 60–90 minutes. All interviews were digitally recorded and later transcribed verbatim into text for analysis, thereby capturing participant views and experiences in their own words.

The interviews explored the key themes of participation in the case study project, as well as individual awareness, behaviour change in electricity use, expectation of electricity bill, impact on community, benefits and barriers to peak electricity demand reduction, and transferability to other regions. These topics were the focus in all interviews, which in turn led to other themes emerging as a consequence of the semi-structured nature of the interviews and the open-ended questioning used. All interviews were conducted by the one researcher.

### Analysis

Transcribed interviews were analysed qualitatively in order to determine patterns or themes pertaining to life or living behaviour [59]. Thematic analysis identified the major issues and topics which emerged from the data. An iterative process was used with the transcripts being read and re-read in order to code the data and identify emerging themes and meaningful categories. The data was manually coded with key themes and sub-themes highlighted, grouped and labelled to enable the creation of a comprehensive observation of participant lived experience and perceptions.

## Results

### From Electricity Bill to Profile in the Community - Topic of Conversation

#### Becoming recognizable

Most residential consumers had little or no contact with the energy utility outside the regular quarterly delivered electricity bill. The case study project involved community based marketing and targeted energy assessments in a majority of residential homes. For some residents a Townsville beach photograph was the initial image use to launch the case study project and a way to introduce the project to the community. The photograph built curiosity about the project in the community and a sense of pride in the community being chosen as the site for this project undertaking.

*“I can tell you one of the best images in my mind is when it was announced we were going to be the Solar City suburb and the picture in the newspaper was “residents spelling out Solar City on the beach” and that is a picture that will remain with me; I was proud that people had done that and we got it.”* House3 Participants

The first representations of the case study project were vivid and commanded community attention, though many participants were initially unaware of what the project was about or what it meant to them individually or as a community. The residents noted that the establishment of regular correspondence with the community provided information and answered fundamental queries about the project.

*“Direct mail; word of mouth, maybe through community news, an ad there; also probably through [local paper] writing about it. I think they had community meetings and things like that. So they tried lots of different avenues and [these] were the best ways of getting people talking about it and getting out there.”* House13 Participants

A major breakthrough at the case study location was making electricity use behaviour part of community conversation.

*“We do talk about power usage with people here on the island; it is a topic of conversation.”* House11 Participants

The energy utility used various strategies to become visible in the community, to develop a profile and to provide greater understanding of the project.

#### Overcoming Cynicism

Those interviewed indicated some cynicism and confusion regarding an energy utility encouraging a reduction of electricity use in the community.

*“There was a lot of cynicism … “what's in it for [the energy utility]?” People couldn't understand why they [the energy utility] were driving down usage on the island. People thought it was pretty irrational for a power supply [the energy utility]. I can understand [the] cynicism.”* House12 Participants

Consumers rarely have contact with energy utilities and potentially only discuss electricity when describing rising electricity prices or a large quarterly electricity bill.

### Profile to Relationship - Changing the Conversation

#### Acceptance by Community Leaders

Residents highlighted the importance of community leaders in building an acceptance of the project and its value to the wider community, by being advocates and providing an expanded line of communication.

*“Once the leaders of the community okayed it and were promoting it, it [case study project] became user-friendly.”* House1 Participants

Local community members facilitated the link between the energy utility, the community and the residents in a variety of ways. Some residents worked directly with the project as paid employees. Having locals working alongside the project team helped develop trust and bring community spirit to the project. These locals were also a positive voice within the community for acceptance and action.

*“If you have got someone who is respected, just as a community member, talking to other people, they just respect them because it's not some government boffin or someone coming in saying, “Oh, this is really good.”.”* House5 Participants

Good practices within the community were developed using local examples of peers and leaders making decisions. The trust within the community translates to encouragement for others once actions were visible.

*“The community is encouraging/supporting each other. We are terribly pleased when one of our mates had their photo taken with panels on the roof behind her … little things like that encouraged others.”* House11 Participants

The influence of community leaders was required to build the network of trust which allows for access and involvement to a greater section of the community. It is this access and involvement which is required for wide scale peak electricity demand reduction.

*“The community involvement, that's what it's all about. You have to involve the community because they are the ones who are going to do it for you. They are the ones who are going to save the money and save electricity, aren't they, or use less electricity. You have to involve them.”* House12 Participants

Success in substantial peak demand reduction can only occur when the majority of the community is fully engaged.

#### Importance of Project Team Makeup

The participants highlighted that choice of the right people to represent the case study project was an essential element to develop a working relationship with the community. The participants viewed the energy utility representatives positively, and seen as important to project success.

*“People here on the team were very keen, passionate/committed. They were a brilliant, working team that operated here, rolling out the program; well-managed and the team itself was enthusiastic and that enthusiasm was one of the reasons for the impact on the community here.”* House9 Participants

The data suggests that the project team can influence the mood of the community, and can attract consumers who may not be early adopters to new endeavours.

*“People want to become recognised and accepted by the [energy utility] team because they were a very positive influence.”* House9 Participants

Many of the interviewees had no prior personal contact with energy utility employees, but explained how the Magnetic Island project team had become part of the community.

*“[Energy utility] became part of the community … to my mind, made a huge difference and I am sure to a lot of people.”* House7 Participants

#### Accessibility

Establishing the “Smart Lifestyle Centre” was identified by most residents as having a significant impact on the integration of the energy utility into the Magnetic Island community. The residents saw the revitalisation of the old community building as an investment in Magnetic Island. Residents explained that the “Smart Lifestyle Centre”, previously a sport and recreation club, as well as musicians club, was revitalised as a social hub for Magnetic Island as well as a place to focus on energy reduction. The centre had been used for several fair days with handymen invited to give energy saving tips to the community and to answer questions on energy use. The centre now holds events and committee meetings for the community, making it an investment well received and respected by local residents. Redevelopment of the community building made a substantial impact in the public conscience.

*“Making that derelict building in Horseshoe Bay usable again - That is phenomenal, really, because that was boarded up and trashed. And they [energy utility] … turned it into something that the community really uses.”* House20 Participants

*“Absolutely one of the most generous/important things that they did for community”* House3 Participants

The Smart Lifestyle Centre provided a site where efficiency equipment could be seen operating, adding to confidence in accepting new technologies.

*“A shopfront where you can go where you can actually see that a low-flow showerhead actually isn't a dribble of water”* House10 Participants

Contact with the electricity industry representatives was a new experience for interviewees, and having them embedded within the community was seen positively.

*“So the community became far more receptive because we are talking to these [energy utility] blokes all the time, with whom we had never spoken to before.”* House1 Participants

The communication within the community formed from the trust and credibility, ready access to the project team, and the profile coverage through Magnetic Island.

*“You can communicate with them easily and talk about them as well; so there's always discussion.”* House12 Participants

*“I think they had community meetings and things like that. So they tried lots of different avenues and were the best ways of getting people talking about it and getting out there.”* House13 Participants

### Relationship to Partnership - Having the Conversation

#### Long term commitment

It would appear from the participant interviews that becoming a peer within a community takes time, consistency and investment by the energy utility. The energy utility was able to establish trust and eventually peer status by being embedded and entrenched over a long period of time.

“[The case study project was] *effective because of the amount of money and the resource put into it and the momentum maintained for a relatively long period of time*.” House9 Participants

“[The case study project was] *well-organised, well-executed and remaining consistent*” House10 Participants

Consistency, longevity and intent on becoming part of the community proved valuable in being accepted as a peer. Many of the participants highlighted the difference between the case study project and other community programmes where a lack of success was often blamed on the limited time given to the community or a narrow objective or agenda.

*“Government is extremely good at going, “We are going to have a new initiative.” By the time it gets running, it runs for 18 months/two years, it is not long enough. I think most importantly is a campaign that lasts long enough. It is not a blow in, blow out.”* House10 Participants

*“It [case study project] didn't just start up and like a lot of things, "Okay, here it is. I have done what I have had to do; now, I am going to take my toys and I am going home.”* House1 Participants

It was important to the community that respect was shown, and the project was not just result driven. In fact, the results indicated that the transition from relationship to partnership can be traced to acceptance within the community and the view that the greater good is part of the relationship.

*“They [the community] feel comfortable with strangers coming in that are going to stay a while and put some benefit into their community … they can see something greater for the community and they will probably embrace it.”* House5 Participants

The success of the energy utility in creating a connection with the community allowed the building of a community culture around electricity demand reduction. The participant interviewees highlighted the presence that the energy utility in the community which helped keep energy efficiency and electricity demand reduction in the conversation and thoughts of the residents.

*“There was constantly this awareness in the background. … It was saturated with [energy utility] stuff.”* House1 Participants

*“Whether it was the public meetings, presentations at public meetings or whether it was the displays that [the energy utility] always did at the market days, you know, there would always be a [energy utility] presence [in the community].”* House13 Participants

#### Community Investment

Investment in the community was often highlighted as a key ingredient in the building of a partnership. The energy utility provided funding and participation in projects, with investments including kids art classes, general art exhibitions, as well as funding local clubs such as the bowls and surf clubs, community planning and many more activities, all of which were cited by residents.

“[Energy utility] *embraced the community and wanted to support the community*.” House3 Participants

This support for the community helped enable the energy utility to move from outsider to insider, given how the interviewees described the relationship.

“[The energy utility] *has been generous. I think they have been very community-minded*.” House5 Participants

The benefits of reduced peak electricity demand may not have been realised directly by the resident in the form of financial reward, but the individual saw benefits of their behaviour change as helping the community, and this was a sufficient trade in their eyes.

*“That would be one of the reasons, too, that I would have liked to support them; the fact that we were getting some other benefits; not necessarily the deduction of electricity, but we had an organisation that was willing to sponsor some of our community … or make even the premises available for community [activities].”* House3 Participants

Investment of profile, credibility and prominence over an extended period of time served to place the project in the minds of the community and as such allowed the energy utility to become embedded, and a member of the community.

*“Look, they were on deck, they were great; they created employment in the area; they gave the electricians on the island jobs to do. As a community member, I was very much looked after and they were consistent; they were thorough, they were professional, they were reliable and they were effective. So overall, they acted with integrity and most of the community were very much in favour of having [the energy utility] there and it was a success.”* House1 Participants

“[Locals] *pride themselves on being community-minded; help your mate …Yeah, “help your mate”, but help within your circle. Don't help some random cause, you know, that is the difference*.” House5 Participants

“[The energy utility] *made themselves part of the fabric of the community*.” House20 Participants

By moving from outsider to insider, the case study community believed in the need to share the problem and combine to help with the solution.

*“I regard this as a shared problem …how do we keep peak [electricity] demand down as time goes on?”* House10 Participants

Overall, the energy utility moved from a quarterly electricity bill to a member of the community, become a peer with the individuals in many households, and formed partnerships to reach objectives and share common goals.

## Discussion

This article presents a qualitative investigation into the success of residential electricity demand reduction within one suburban community. This demand reduction across the residential community examined over an extended period of time indicates that the case study project was successful at engaging with the community and promoting energy efficiency and behaviour change of electricity use (see Appendices A and B). Analysis of the interviews illustrated the importance of establishing a relationship between the energy utility, the community and the residential consumers. The embedding of an energy utility within the social network of the community created the opportunity to educate, influence and to affect demand reduction. This assisted the energy utility to transform from bill sender (seen as a transaction) to peer and partner of individuals in the community such was the acceptance that utility representatives were invited into the majority of residential households (greater than 80%) to undertake home energy assessments. The following section discusses the major factors, identified by the participants, in establishing the peer-community relationship.

### Peer Acceptance and Partnership

A partnership requires commitment and trust, and not just a one-sided dependence. Successful partnerships have partners who respect one another, and the participants reported very positively about the energy utility. At some stage during the case study project, the energy utility became a peer with individuals in the community. The cohesion of the relationship determined peer acceptance, with social communication becoming more frequent between residents and the utility. The participants indicated that the collaborative alliances established between them (the residents) and the energy utility facilitated positive relationships, and helped influence the community as a whole to reduce peak electricity demand. Mutual benefit was often discussed as a means for behaviour change. Although for residents, peak electricity demand reduction was not necessarily a benefit for the individual, participants indicated a desire to cooperate with the utility due to the peer-community relationship.

It was open communication with the residents that moved the conversation from “what about me” to “what can I do”. Once the partnership developed to this level, all parties signed-up as members of the behaviour change team and this community membership was valuable in reaching goals. The findings indicate that when the community can see investment for the common good, individuals are more interested in participating in activities which have no self-interest. The results of this research would seem to show that peer acceptance and partnership development is beneficial to fully elicit appropriate behaviour change. This observation is backed by research that the individual is more likely to change behaviour or habits with peer influence than financial gain because of the desire for social approval from behaviour change [60].

Successful acceptance as a peer and building a community partnership was attributed to the three major influences - trust and credibility, the acceptance and support of community leaders, and acceptance of the energy utility by the community. The three constructs of a) value of trust and credibility, b) influence of community leaders, and c) becoming a member of a community, which formed the basis of peer acceptance and partnership for an energy utility at the case study location, are explored below.

#### The Role of Trust and Credibility

The participants spoke of cynicism in the early stages of the energy utility presence in their community. Therefore, one of the early challenges for the utility was presenting their objectives and motives. Confusion on why an energy utility, who makes profits from people using electricity, would want the community to reduce electricity demand was difficult to understand, and this premise became the topic of debate amongst many community members.

This negative reaction was addressed by the energy utility interacting with the community, by listening to community concerns, explaining the energy utility direction through clear communication, and by presenting a physical presence in the community to increase accessibility and promote further conversation. All four factors not only overcome cynicism of the energy utility motives, but also helped develop empathy through regular communication and build a relationship with the community. The trust of the community allowed a partnership between the energy utility and the community to mature.

The participants saw the energy utility as a trustworthy member who worked with and supported the residents and the community more broadly. This trustworthiness spread throughout the Magnetic Island community and was considered important, by participants, for the relationship between industry and households. Explaining the potential for peak electricity demand reduction to the Magnetic Island residents required the energy utility to move from supplier of a quarterly electricity bill to a higher order connection with the individual electricity consumer. An essential element is the trust that the householder placed in the energy utility representative. When a person believes they are not knowledgeable in an area, such as the resource efficiency of their house, they are most likely to be persuaded by someone whom they believe is credible. This means given the lack of knowledge that householders have regarding home resource use, the perceived credibility of the utility representative, built from accessibility and regular interaction, plays a crucial role in determining what, if any, action is taken to reduce peak electricity demand.

#### Influence of Community Leaders

Another essential element in building a partnership was acceptance by community leaders, as these members of the community are at the grassroots of local opinion. Although each residential electricity household received an electricity bill at least every three months, there was little knowledge or contact with the electricity industry. Essentially as an outsider, early acceptance by community leaders was seen by participants as a foundation step in general acceptance by the community residents. This general acceptance by residents translated to the opportunity to build relationships and partnerships with the community and the individuals within. Successful collaborations with community leaders helped accelerate the relationship and broaden the conversation for electricity use behaviour change. Further, developing alliances within the community allowed greater access to many more residents due to peer influence, therefore broadening community engagement.

The peer-community relationship developed through this community engagement became an enabler in energy efficiency improvements and behaviour change. As such, the community was empowered to participate in the goal of reducing peak electricity demand, and to consider making the necessary changes to their individual behaviour. The encouragement by community leaders, collaborating with the energy utility, gave impetus to the residential consumers taking action with the belief that their actions could successfully address the peak electricity demand issues in the community. The energy utility was seen as sharing responsibility with the residents across a broad spectrum of activities within the community.

The influence of community leaders allowed for greater emersion into the community fabric, with early adopter community members reinforcing the messages by the energy utility and showing that behaviour change was acceptable in the first place and desirable outcome from the community perspective. Therefore, community leaders helped accelerate peer acceptance of the energy utility by the community.

#### Becoming a Member of the Community

Becoming part of the community through acceptance occurred when the energy utility was seen to care and respect the views of the community. This care was shown by many aspects of community presence such as the Smart Lifestyle Centre and regular participation in social activities, as well as investment and various financial contributions in local sporting clubs and community initiatives. To become a community member, the energy utility needed to understand the culture and nuance of similarities and differences with individuals.

Contact with energy industry representatives is uncommon and the energy utility on Magnetic Island only became embedded when its representatives became familiar to the local population. The presence of the energy utility reduced concerns participants had indicated about government agencies often having short term goals. The need to become locally based within the community, and fully accessible to individuals, helped address this previous distant relationship of electricity provider and bill sender. The choice of passionate and knowledgeable project team members was essential to the success of the project and created a positive face of the energy utility in the community. The team needed to integrate with the local population, show patience and understanding as the credible expert in electricity use behaviour change, and provide personalised information to assist in peak electricity demand reduction. The participants indicated that listening to the needs of the community, and investing time with the residents rather than focusing totally on immediate action or energy utility final goals, was received very positively and a key factor in establishing a peer-community relationship.

Communication on successful community goals also assists the continuation of behaviour change and the acceptance of responsibility. Success can be a contributing factor in the ongoing behaviour change and the further reduction of peak electricity demand and total energy consumption. Residential consumers saw their contribution as valuable individually, for the community as a whole, and for the utility. Local media reporting of reduced peak electricity demand and total consumption broadened the audience and extended the success. Communication on successful community objectives and goals also helped assist continuation of behaviour change and the acceptance of responsibility.

### Policy Implications

The findings of this research would indicate that relationship building between energy utilities, communities and residential consumers may provide opportunity to limit ever-increasing infrastructure costs for the electricity industry. Given that the case study project was able to perform energy assessments in more than 80% of households suggests that the project has reached a greater population than only residents in the community who are “inclined to change” or those with a conservation ethos. This population coverage could be of interest to policy designers who are looking for mass residential involvement in energy demand reduction.

## Conclusion

Embedding the utility into the community, to becoming a peer and partner appears to provide a foundation to deliver significant reduction to peak electricity demand. To firstly reduce peak electricity demand, and then to sustain the peak electricity demand reductions for an extended timeframe is rare. At the case study region, the peak electricity demand in summer 2011 was below the peak electricity demand prior to case study project implementation in summer 2008.

Becoming a peer and developing partnerships within the community acted as a catalyst for successful change in energy use behaviour practices. This research implies that all government agencies could rethink their relationship with the community, and revert to a community-based approach when undertaking new mass population objectives. This research highlighted that community and individuals can embrace change for the common good as well as for personal gain. Addressing objectives such as low carbon use or sustainability could apply this peer-community approach to implement meaningful large scale energy efficiency and electricity use behaviour change. The possibilities for promoting energy conservation as well as pro-environmental behaviour through reduction of energy and water use, as well as reducing waste and limiting emissions, would lead to comprehensive benefits to the current and future population.

## Supporting Information

Appendix S1 (DOCX)
