# Supplementary material for: Moving from Outsider to Insider: Peer Status and Partnerships between Electricity Utilities and Residential Consumers
Source: PLoS One. 2014 Jun 30;9(6):e101189. doi: 10.1371/journal.pone.0101189 (PMC4076285; doi:10.1371/journal.pone.0101189)
Supplement: Appendix S1 — (DOCX) [file pone.0101189.s001.docx]

# Appendix S1

Magnetic Island is eight kilometres off the coast of North Queensland and is considered a suburb of Townsville with commuting workers, as well as being a desirable retirement location and popular holiday destination. The permanent population is approximately 2200, although this fluctuates in holiday times. The majority of customers (more than 99%) on Magnetic Island are non-market customers who are charged regulated residential electricity prices set by the Queensland Competition Authority under their delegated powers from the Queensland Government.

Magnetic Island has clear geographical and electricity network boundaries which make it an easily definable region for research and analysis purposes. Magnetic Island was selected as the site for the Townsville Solar City Project because of the need for a third undersea cable, within the immediate planning horizon, due to increasing peak electricity demand forecasts. The Townsville Solar City Project was part of the Australian Government National Solar Cities Partnership between all levels of government, industry, business and local communities to trial sustainable energy solutions and to rethink the way Australia produces, consumes and conserves electricity.

The Townsville Solar City Project was designed on two key principles of Community-Based Social Marketing [[1](#_ENREF_1)], and Thematic Communication [[2](#_ENREF_2)]. Community-Based Social Marketing removes of barriers and enhances of benefits to behaviour change at the community level [[1](#_ENREF_1)] while the second design principle of Thematic Communication inspires and provokes consumer thoughts, while being entertaining and enjoyable, easy to understand, and relevant to the consumers [[2](#_ENREF_2)].

The Townsville Solar City Project installed 720kW of solar photovoltaic panels (PVs) to the two feeders on Magnetic Island with the output supplying up to 25% of the island load. All electricity from the PVs is fed back into the grid and the ownership and maintenance of the PVs remains with the energy utility company. Residential households allowed the PVs to be installed on their roof, for collective benefit and without individual gain. The electricity from the PVs does not reduce peak demand on the island due to the times of peak being after sunset.

The Townsville Solar City Project highlighted benefits and removed barriers to electricity demand reduction for Magnetic Island residents. Some examples include the installation of energy efficient light bulbs and showerheads, cash-back vouchers for upgrading to energy efficient appliances or reflective roof paint, the removal of old inefficient appliances from the home, the use of tariffs to avoid peak demand times, and the use of prompts to remind the consumer to set air-conditioners to 25degC, lower water heater temperatures, or take shorter showers. The Townsville Solar City Project has conducted 1425 residential energy assessments, out of a total of 1735 residential households on Magnetic Island, since February 2008, representing greater than 80% of the residential homes on Magnetic Island.

Results from the Townsville Solar City Project are shown in Figures S1 and S2 below. From Figures S1, the peak electricity demand for 2011 was lower than the peak level reached prior to the implementation of the Townsville Solar City Project in 2008. From Figure S2, it can be seen that the grid connected electricity consumption by 2011 was below electricity consumption levels in 2005.

**Figure S1 – Daily Peak Electricity Demand at Magnetic Island [**[**3**](#_ENREF_3)**]**


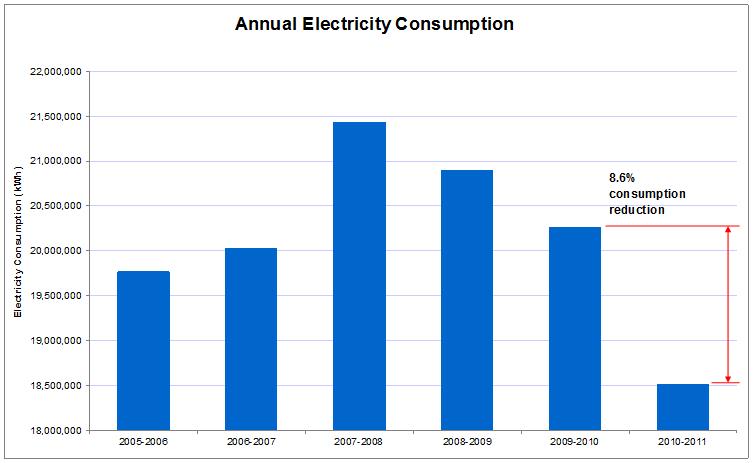
 **Figure S2 – Grid Supplied Annual Electricity Consumption at Magnetic Island [**[**3**](#_ENREF_3)**]**

# Reference List

1. McKenzie-Mohr D (2011) Fostering sustainable behavior: An introduction to community-based social marketing. Gabriola Island, Canada: New Society Publishers.

2. Ham SH (1992) Environmental interpretation: A practical guide for people with big ideas and small budgets: Fulcrum Publishing.

3. Townsville Solar City Project (2012) Townsville Queensland Solar City Annual Report. In: Government Q, editor.
